# Graded Effects of Social Conformity on Recognition Memory

**DOI:** 10.1371/journal.pone.0009270

**Published:** 2010-02-17

**Authors:** Nikolai Axmacher, Anna Gossen, Christian E. Elger, Juergen Fell

**Affiliations:** Department of Epileptology, University of Bonn, Bonn, Germany; University of Sydney, Australia

## Abstract

Previous studies have shown that the opinion of confederates in a group influences recognition memory, but inconsistent results have been obtained concerning the question of whether recognition of items as old and new are affected similarly, possibly because only one or two confederates are present during the recognition phase. Here, we present data from a study where recognition of novel faces was tested in the presence of four confederates. In a long version of this experiment, recognition of items as old and new was similarly affected by group responses. However, in the short version, recognition of old items depended proportionally on the number of correct group responses, while rejection of new items only decreased significantly when all confederates gave an incorrect response. These findings indicate that differential effects of social conformity on recognition of items as old and new occur in situations with an intermediate level of group pressure.

## Introduction

Long-term memory as the basis for the adaptation of human behavior to experiences is crucial for survival, but far from being perfect [1], [2]. Thus, various kinds of remedies – from complex strategies and mnemotechniques to simple notes on scratchpads – are used to help against forgetting [3]. In addition, social peers who experienced the same event can be asked for their opinion. The drawback of this habit is of course that even correct memories can be negatively influenced by peers. Since the pioneering studies of Asch [4], the impact of peer pressure on cognitive functions has been extensively studied [5]–[7], and recently also the neural basis of social conformity has been investigated [8], [9]. Peer-group effects on memory are particularly important because of the legal relevance of eyewitness testimony [10], [11]. While majority decisions are usually more accurate than those of individual subjects [12], multiple individuals in a group perform worse in free recall tasks than the joint individuals, probably due to a disruption of retrieval strategies [13]–[15].

While these studies focused on free recall of previously encoded items, other designs investigated the impact of confederates' responses on recognition memory. Schneider and Watkins [16] tested recognition memory for word lists with two participants responding loudly “old” or “new” after presentation of each item in the recognition phase. They found that the response of the first participant strongly influenced the response of the second participant. This finding was replicated by Reysen [17] with a virtual confederate, who in addition found that in a subsequent individual testing session, participants still tended to respond according to the previously seen group opinion, suggesting that group opinion actually implants new memories. Similar effects of social conformity were observed when photographs of cars were presented [18].

While these studies convincingly demonstrate the effects of social conformity on recognition memory, they differed from the seminal study of Asch [4] in that only a single [16]–[19] or two [20] confederates participated. Therefore, the normative effects of group majorities cannot be distinguished from the effects of individual subjects. Moreover, it has been shown that subjects are more likely to adhere to their own opinion if at least a single confederate disagrees with the majority (dissenter effect; [4]). This effect is most likely higher in the case of a relatively simple task: Although task difficulty does not influence normative group pressure, the informational relevance of the majority opinion is less relevant if subjects are more certain about their individual decisions [21], [22], especially if the task is considered relevant [23].

**Figure 1:**
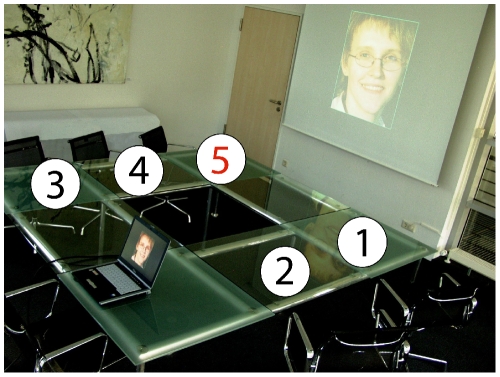
Experimental procedure. Four confederates, seated at positions 1–4, one participant, seated at position 5, and the experimenter, seated in front of a laptop, participated in the experiment. During encoding and retrieval, figures of unknown female and male faces were presented. During retrieval, all confederates and the participant loudly rated each face as either “old” or “new”, and all responses were documented by the experimenter. Importantly, all confederates gave their responses prior to the test participant.

Here, we studied the effect of a group of four confederates on recognition memory (see Fig. 1 for a photo of the experimental setting). We hypothesized that participants would be more likely to respond according to the group if more confederates exerted implicit pressure on them (see [20], where effects of one and two confederates were compared). To maximize effects of social conformity, participants responded in the presence of the confederates, because conformity is significantly larger during public responses [5]. To investigate effects of social conformity as a function of task difficulty, two versions of the experiment were conducted, containing 75 and 150 items during encoding, respectively.

## Results

**Figure 2:**
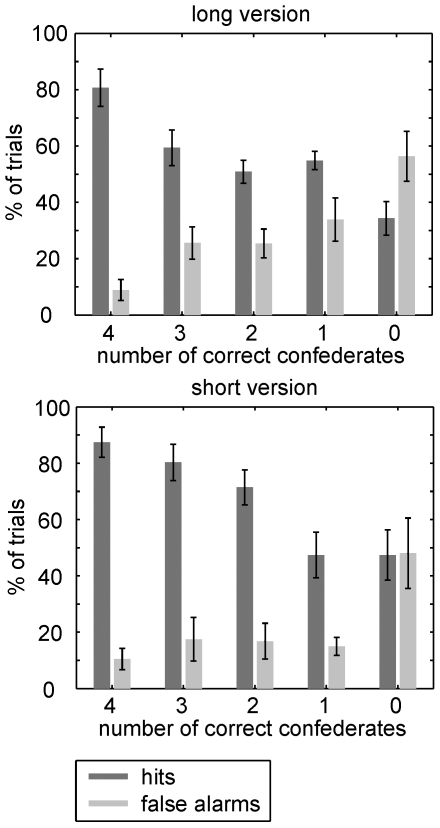
Conformity effects on memory. Dark gray bars indicate percentage of hits (i.e., correct responses during presentation of old items), light bars percentage of false alarms (i.e., incorrect responses during presentation of new items). Bars are normalized to the total number of items presented in each group response condition, separately for old and new items. Memory was significantly better than chance and was affected by group opinion (“memory”×“group” interaction), indicating a highly significant effect of conformity. This effect was similar for the long and short version of the experiment. Error bars indicate s.e.m.

We first analyzed whether memory was better than chance, and whether it depended on either the responses of the confederates or on the version of the experiment. We calculated a three-way ANOVA with “memory” (hits vs. false alarms) and “group” (number of correct responses in the group, ranging from 0 (when all confederates claimed that an old item was in fact new, or that a new item was old) to 4 (when all confederates gave a correct response)) as repeated measures and “version” (long vs. short) as independent variable. The results are depicted in Fig. 2. We found a significant effect of “memory” (F_1,17_ = 90.340; p<0.001), indicating that performance was much better than chance, i.e. that there were more hits than false alarms. Besides, there were significant interactions of “memory” × “group” (F_4,68_ = 24.96; p<0.001; Huynh-Feldt ε = 0.726), demonstrating that memory depended significantly on the confederates' responses; of “memory” × “version” (F_1,17_ = 7.106; p<0.05), indicating that memory was significantly better in the short version; and of “group” × “version” (F_4,68_ = 3.598; p<0.05; ε = 0.844), showing that group effects were different in the long and short version. However, the lack of a three-way interaction showed that group responses had similar impacts on memory in both versions (F_4,68_ = 0.393; p = 0.752; ε = 0.726).

Because items were either rated as “old” or as “new”, the sum of hits and misses in each of the different conditions defined by the group response equaled 100 %, i.e. the number of hits and misses is interdependent. Thus, we could not differentiate between the participants' memory performance for old and new items with the above analysis. To investigate differential effects for old and new items, we next calculated an ANOVA based on correctly recognized old and new items. This analysis contained the repeated measures “item type” (hits vs. correct rejections) and “group”, and the independent variable “version”. Indeed, we observed main effects of “item type” (F_1,17_ = 16.01; p<0.001), indicating that memory performance was significantly better for new items than for old items; of “group” (F_4,68_ = 24.95; p<0.001; ε = 0.726), showing that memory decreased significantly with group; and of “version” (F_1,17_ = 7.121; p<0.05), because memory performance was worse in the long than in the short version. Moreover, we observed a significant three-way interaction (F_4,68_ = 3.600; p<0.05; ε = 0.843), indicating different “group” × “item type” interactions in the long and short version.

To further elucidate these effects, we calculated two-way ANOVAs with the repeated measures “item type” and “group” separately for the short and long version. For the long version, we observed main effects of “item type” (F_1,9_ = 33.046; p<0.001), indicating that there were more new items which were correctly rejected than old items which were correctly recognized, and of “group” (F_4,36_ = 12.928; p<0.001; ε = 0.762), showing that conformity affected both items types. However, there was no “group” × “item type” interaction (F_4,36_ = 1.796; p = 0.162; ε = 0.866), indicating similar conformity effects on the processing of old and new items.

For the short version, results were strikingly different: While there was also a main effect of “group” (F_4,32_ = 12.373; p<0.001; ε = 0.821), the main effect of “item type” did not reach significance (F_1,8_ = 1.966; p = 0.199). However, there was a significant “group” × “item type” interaction (F_4,32_ = 5.135; p<0.005; ε = 0.936), demonstrating different group effects on the processing of old and new items.

Separate one-way ANOVAs for old and new items in the short version revealed group effects for both item types (old items: F_4,32_ = 11.404; p<0.001; new items: F_4,32_ = 8.856; p<0.001). However, subsequent t-tests showed different patterns of group effects for old and new items (Table 1): For hits, there were significant differences between various levels of group conformity; for correct rejections, only the “zero correct” group condition (in which all confederates gave an incorrect response) affected memory performance.

**Table 1:** Conformity effects in the short version.

	# correct responses	3	2	1	0
**Old**	**4**	t = 1.76 p = 0.12	t = 4.44 p = 0.002	t = 5.69 p<0.001	t = 5.13 p<0.001
	**3**		t = 1.61 p = 0.15	t = 3.01 p = 0.02	t = 3.20 p = 0.01
	**2**			t = 2.69 p = 0.03	t = 2.50 p = 0.04
	**1**				t = 0.07 p = 0.95
**New**	**4**	t = 1.98 p = 0.08	t = 1.67 p = 0.13	t = 1.62 p = 0.14	t = 3.56 p = 0.007
	**3**		t = 0.22 p = 0.83	t = 0.05 p = 0.96	t = 3.69 p = 0.006
	**2**			t = 0.24 p = 0.82	t = 3.76 p = 0.006

This table depicts the results of pair-wise t-tests for all group response conditions in the short version of the task, where social conformity exerted different effects on old and new items. While there were significant differences between various conditions for old items, there were only significant differences in the “zero correct” condition for new items.

The responses to the questionnaire are depicted in Table 2. The first two questions concern the accuracy of one's own and the others' responses. To statistically compare the results, response 1, “often correct” was set as “3”, response 2, “sometimes correct”, as “2” and response 3, “rarely correct”, as 1. Participants in the long version estimated their own accuracy as high as participants in the short version (t_17_ = 0.520; p = 0.610; question 1). Confidence in the other participants' responses was not altered neither (t_17_ = 1.370; p = 0.19; question 2), and the subjectively perceived influence by other participants was similar (both in the long and in the short version, 3 participants indicated to perceive an influence; question 3). Of those who did respond to question 4 (“Did you ever conform to the decision of the other participants?”), most participants indicated as a reason that they did so because they were not sure; importantly, only a single out of 19 participants indicated to respond according to the others to conform with the majority, strongly suggesting that conformity was not due to consciously perceived social pressure (although it does not argue against unconscious normative influences, of course). Finally, in question 5 (“Did you ever decide against the majority?”), most participants (16 out of 19) selected item 1 (“You were sure that your response was correct, and did not mind the response of the others”).

**Table 2:** Results of the questionnaire.

Experiment version	long	long	long	short	short	short
**Questionnaire response**	**1**	**2**	**3**	**1**	**2**	**3**
**How do you judge the accuracy of your responses?**(1) Often correct/(2) Sometimes correct/(3) Rarely correct.	2	7	1	2	7	0
**How do you judge the accuracy of the other subjects' responses?**(1) Often correct/(2) Sometimes correct/(3) Rarely correct.	0	9	1	3	5	1
**Did you feel influenced by the other subjects' responses?**(1) Yes/(2) No.	3	7		3	6	
**Did you ever conform to the decision of the other subjects? If so, please indicate why.**(1) You were sure that your response was correct, and the others responded equally.(2) You were sure that your response was correct, but responding according to the others to conform with the majority.(3) You were not sure and therefore conformed with the majority.	1	1	6	2	0	5
**Did you ever decide against the majority? If so, please indicate why.**(1) You were sure that your response was correct, and did not mind the response of the others.(2) The others' responses made you feel unsure, but you still maintained your opinion.(3) You considered the responses of the others incorrect.	8	3	2	8	1	0

The table depicts the number of participants choosing the different response items in the two experiment versions.

## Discussion

We investigated the effects of social conformity on recognition memory for faces in a paradigm with four confederates. This task was conducted both in a long and a short version to test conformity effects as a function of the difficulty of a memory task. In the initial study by Schneider and Watkins [16], participants responded partly before and partly after a confederate (or a second participant). The response rates during the trials where the participants responded first were taken as baseline. However, it should be noted that conformity effects may still play a role in this baseline condition: For example, participants may be tempted to bias their responses either toward “old” or “new” responses; this might be dependent on previous responses by the other participant. In our experiment, even in the condition where the responses of the confederates cancel out (two confederates responding “old” and two responding “new”), the participant's response is possibly biased due to the mere presence of the group. To exclude possible effects of the group situation, we did not assume a baseline for memory responses. Similar to our approach, Reysen [17], [20] directly compared rates of hits and correct rejection without assuming a baseline.

We found stable effects of memory, indicating that participants could reliably differentiate old and new items, but also “memory” × “group” interactions, showing that responses were significantly affected by the confederates (Fig. 2). The influence of social conformity depended on task difficulty and varied between old and new items: In the long version, memory was significantly worse than in the short version, and group responses affected individual responses towards old and new items similarly. In the short version, however, there was a different impact on old and new items: While the number of hits decreased significantly as soon as two confederates gave incorrect responses (Table 1), the rate of correct rejections remained high (>80%) as long as a single confederate gave a correct response. Thus, in this short version of the task, correct rejections of new items were less susceptible to the confederates' responses as long as they were non-uniform.

This result appears to be in contrast to findings of Reysen [20] who investigated the influence of two confederates on recognition of words. He found that while the rate of correct rejections decreased significantly if one of these confederates gave an incorrect response (as compared to both responding correctly), there was no further difference when both confederates responded incorrectly (as compared to when their opinion was divided). However, several differences between our study and the study by Reysen [20] should be taken into account. First, in our study, four instead of two confederates participated. Thus, the impression of a group of four participants giving a uniform response is likely stronger than if only two participants give the same response. Second, in the study by Reysen [20], familiar words instead of novel unknown faces were shown as stimuli. Thus, rejection of new items is likely due to different mechanisms; in our study, detection of perceptual novelty is sufficient to categorize an item as new, while it requires distinction of recently seen versus not-recently seen familiar words in Reysen's [20] experiment.

Previous studies reported that conformity effects were larger for new than old items; in other words, that participants are more likely to have false memories, i.e. to consider new items for old, than to forget old items, i.e. to believe that actually old items are new [19]. In contrast, we found that conformity effects on hits were significantly stronger than on correct rejection. This may be explained by the fact that we used four confederates, so that the impact of group responses was parametrically scaled. Indeed, the difference between the “four correct” and the “zero correct” condition in the short version was highly significant for both old and new items (Table 1: old items: t_8_ = 5.13; new items: t_8_ = 3.56). Differential effects between old and new items became only apparent when intermediate conformity effects (corresponding to non-uniform responses of the confederates) were taken into account, which could not be investigated in the study of Wright, Mathews and Skagerberg [19] with only one confederate. Thus, some effects of social conformity might only become apparent if multiple confederates participate in an experiment.

Why did we observe more pronounced effects of social conformity on recognition of old as compared to new items? The fact that this difference became only apparent in the short, but not in the long version suggests that effects of task difficulty play a role here. In the three-way ANOVA across both versions, we observed a main effect of “item type”, indicating that detection of new items was better than recognition of old items; in the short version, the rate of correct rejections was not significantly affected by group opinion as long as at least one out of four confederates gave a correct responses. Thus, participants were apparently very sure that they did not see the new picture before, so that they ignored the responses of the confederates. The findings in the initial study by Schneider and Watkins [16] rather resembled the results of the long version of our experiment: Even though they observed a generally higher rate of correct rejections (82%, 75%, and 59% in the case of a correct previous response, no previous response, and an incorrect previous response) than of hits (74%, 62%, and 55%), social conformity was similar in the two conditions.

These results suggest that previous findings that social conformity affects detection of new items stronger than recognition of old items [19] cannot be easily generalized to real-world conditions. More specifically, our results show that rejection of new items may be actually more accurate (and thus less susceptible to social conformity) than recognition of old items if stimuli are novel, rather distinct and participants are capable of distinguishing subtle details, as is the case for human faces. In this situation, some small divergence in the opinion of other participants might suffice to rely on a correct personal opinion.

One limitation of our study is that we did not conduct an additional memory test in the absence of the group (as in, e.g., [17]) or include a condition where the test participant responded prior to the confederates (as in, e.g., [16], [18]). In the responses to the questionnaire, only one of 19 participants indicated to respond according to the group in order to conform to the majority. This strongly suggests that group influence was not due to subjectively perceived social pressure, but indeed involved a feeling of familiarity with or novelty of an item. However, we cannot distinguish between the possibilities that (1) the participants' responses are based on actual alterations of memory traces, or that (2) these responses are based on erroneous feelings of familiarity/novelty without modifications of the memory traces. Results from previous studies suggest that conformity to a confederate did indeed result in modifications of the memory trace: Participants performed better in an individual recognition test when they had before responded prior to a (virtual) confederate than when they had responded following this confederate [17]. Similarly, during free recall, participants erroneously recalled items individually that were previously falsely suggested to them by a confederate [24]. This effect even persisted when participants were explicitly warned about the possibility of an incorrect response by the confederates. These results suggest that conforming to a confederate is actually likely to alter memory traces; however, this effect might be graded in the case of multiple confederates. In the current study, we mainly aimed at establishing a situation where a relatively large group of four confederates were present together with the test participant. Further studies will be necessary to identify the exact processes influencing participants' responses in the presence of multiple confederates.

## Materials and Methods

### Ethics Statement

The study was approved by the local ethics committee (“Ethikkommission an der Medizinischen Fakultät der Rheinischen Friedrich-Wilhelms-Universität Bonn”), and all participants provided written informed consent.

### Participants, Design, and Materials

Nineteen participants recruited at the University of Bonn via placard participated in the study (age, mean ± std.: 25.2±3.5 years, 13 females). We used a 2×2×5 mixed design with the between-subject variable “experiment version” (short vs. long) and the repeated measures “item type” (old vs. novel) and “group opinion” (0 to 4 correct responses by the confederates). The experiment consisted of a relatively short encoding and a longer, self-paced recognition phase. During encoding, participants were presented 50 (short version) or 100 (long version) color images of unknown male and female faces from a large database (presentation time: 2000 ms; no inter-item interval). Pictures were pseudo-randomly assigned to the different conditions (old or new). Three different versions were used in each the long and the short condition of the experiment to exclude that by chance more distinctive faces were used as old items or new (distracter) items, thereby introducing a difference between these conditions. After the encoding part, there was a break of 5 min duration during which participants filled out forms (contact information, bank account, etc.) and read a detailed instruction for the retrieval part. During retrieval, participants were presented the old items randomly intermixed with an equal number of new folds; timing of stimulus presentation was self-paced during this phase.

### Procedure

Participants arrived at the laboratory alone, randomly intermixed with four confederates who were recruited from the laboratory personnel. Only students without prior knowledge of the laboratory participated in the study to exclude revelation of the delusive mandate of the confederates. Upon arrival, participants were seated around a table carrying name tags, with the test participant positioned at one end of the table (Position “5” in Fig. 1). After signing an informed consent, participants were instructed that they participated in a study designed to investigate recognition memory for faces and which consisted of both an encoding and a retrieval phase. It was explained that, after encoding and the 5 min break, all items would be presented again randomly intermixed with new items and that each item would be visible until each participant loudly responded “old” or “new”. The order of responses was determined to be clockwise, with all four confederates responding prior to the test participant. During retrieval, the confederates in fact responded to thin colored boxes around the figures. Between zero and four confederates responded correctly, and an equal number of “old” and “new” responses were given to items which were actually old and new. Pictures remained visible until the test participant gave a response. The total duration of the recognition phase was about 20 min. in the short and 40 min. in the long version of the experiment. After completion of the experiment but prior to debriefing, all participants filled out the questionnaire described in Table 2. Then, the test participant was debriefed about the true purpose of the experiment. This was done in the absence of the confederates to avoid an unpleasant situation for the test participant.

### Statistics

P-values in the ANOVAs were Huynh-Feldt corrected for inhomogeneities of covariance when necessary [25].
